# *Enterococcus hirae* bacteremia associated with perinephric collection and renal abscesses in a diabetic woman

**DOI:** 10.1093/omcr/omac101

**Published:** 2022-09-26

**Authors:** Chee Yik Chang, Mogeshwari Jayabalan, Yi Lung Gan, Anuradha P Radhakrishnan, Edmund L C Ong

**Affiliations:** Medical Department, Hospital Selayang, Batu Caves, Selangor, Malaysia; Gombak District Health Office, Batu Caves, Selangor, Malaysia; Medical Department, Hospital Selayang, Batu Caves, Selangor, Malaysia; Medical Department, Hospital Selayang, Batu Caves, Selangor, Malaysia; University of Newcastle Medical School, Newcastle upon Tyne, UK

## Abstract

*Enterococcus hirae* infection accounts for about 1% of all enterococcal infections. This number is likely to be underestimated because of inadequate identification. Human infection due to *E. hirae* is rarely reported. We present the case of a young woman with diabetes mellitus who developed symptoms of pyelonephritis and diabetic ketoacidosis. Renal computed tomography scan revealed the presence of subcapsular perinephric collection and renal abscesses. Her blood culture yielded *E. hirae*. Our patient was successfully treated with antimicrobials based on the susceptibility result. To our knowledge, this is the first reported case of perinephric collection and renal abscesses associated with *E. hirae* bacteremia.

## INTRODUCTION

Enterococci are Gram-positive facultative anaerobic cocci that is rarely implicated in human disease. *Enterococcus faecalis* and *Enterococcus faecium* are the most prevalent species cultured from humans [1]. *E. hirae*, a species belonging to the Enterococcus is a zoonotic pathogen and rarely isolated from human infections [2]. Despite the rarity of *E. hirae* infection in humans, it is known to cause serious and life-threatening illness [3]. Clinicians need to be more aware of the clinical significance of this unusual pathogen.

## CASE PRESENTATION

A 21-year-old woman with poorly controlled type 1 diabetes mellitus (HbA1c = 15%) presented with a three-day history of fever, dysuria, and flank pain. She denied hematuria, urinary frequency, or urge incontinence. Physical examination revealed that the patient was alert, febrile, and hemodynamically stable. The oxygen saturation was 98% as measured by pulse oximeter while breathing ambient air. There was costovertebral angle tenderness on the left side. The remainder of the physical examination was unremarkable.

Hematological analysis revealed a white blood cell count of 23.4 × 10^9^/l (normal range, 4–11 × 10^9^/l) with a neutrophil percentage of 85%, a hemoglobin of 11.8 g/dl (normal range, 12–16 g/dl), and a platelet count of 329 × 10^9^/l (normal range, 150–400 × 10^9^/l). The random blood glucose was 22.2 mmol/l, and a venous blood gas revealed metabolic acidosis (pH: 7.15, serum bicarbonate: 12.2 mmol/l, lactate: 2.3 mmol/l, and anion gap: 21.8 mmol/l. A urinalysis revealed ketonuria (4+) and glycosuria (2+) but leukocyte and nitrite were absent. The chest and abdominal radiographs as well as transthoracic echocardiography were normal. A workup for tuberculosis was negative.

**Figure 1:**
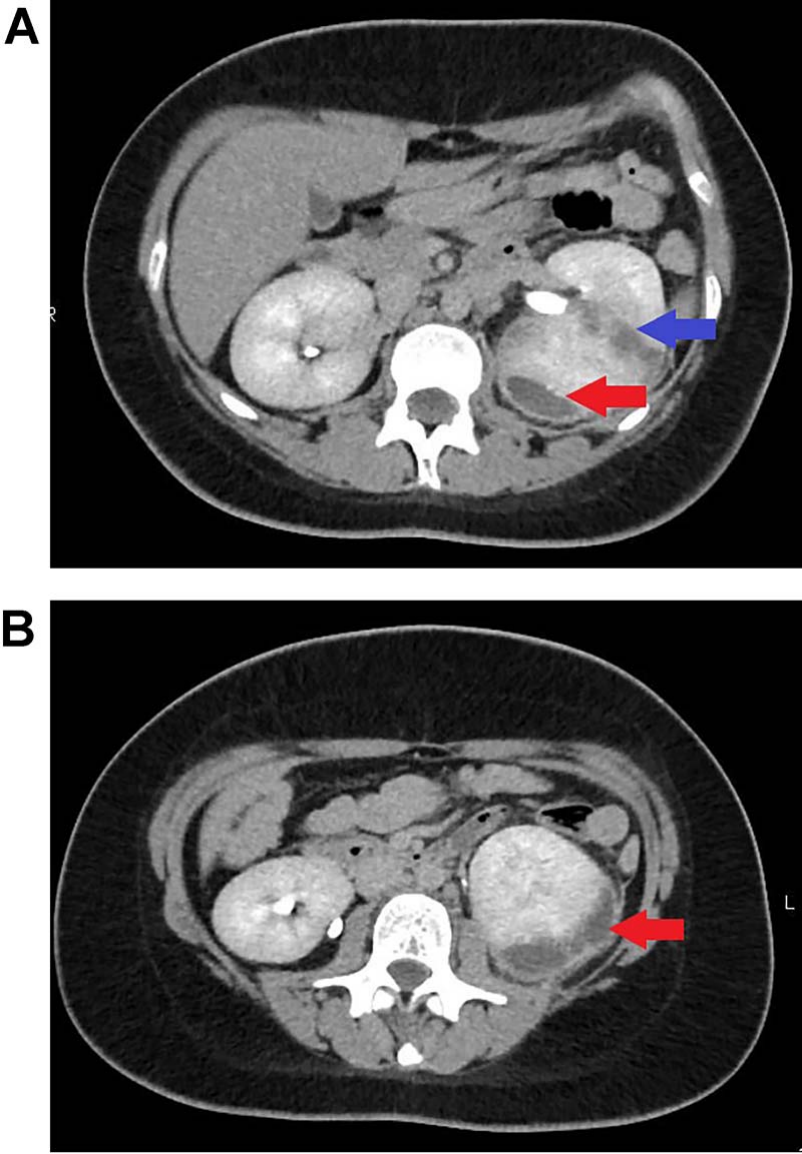
(**a**) Renal CT scan showing an abscess in the left kidney (blue arrow) and subcapsular perinephric collection (red arrow). (**b**) Renal CT scan showing subcapsular perinephric collection in the left kidney (red arrow).

The patient was diagnosed with diabetic ketoacidosis (DKA) and concurrent fluid resuscitation and insulin therapy were instituted. DKA resolved within 24 h. However, the presence of fever, which is not a symptom of ketoacidosis, as well as loin pain and tenderness, prompted us to rule out renal or perinephric infection. Hence, a renal 4-phase computed tomography (CT) scan was performed, which revealed a left subcapsular perinephric collection measuring 1.9 × 4.7 × 9.1 cm, as well as multiple small renal abscesses at the upper and midpole of the left kidney, which coalesced with the subcapsular collection (Fig. 1). Both kidneys were normal in size, and neither had a calculus, hydronephrosis, or hydroureter.

Percutaneous ultrasound-guided aspiration of the left perinephric collection was performed in which 10 cc of purulent material was aspirated. The patient was treated empirically with intravenous ceftriaxone 2 g once daily. After 5 days of incubation, the blood culture was positive for *E. hirae*, using the matrix-assisted laser desorption ionization time-of-flight mass spectrometry (MALDI-TOF MS) method. The blood culture isolate was susceptible to ampicillin, penicillin, and gentamicin, but cultures of the urine and purulent material were negative. Upon receipt of the blood culture result, the antibiotic was deescalated to intravenous ampicillin-sulbactam 3 g every 6 h after received 5 days of ceftriaxone. She showed marked clinical improvement in which the fever and flank pain subsided as well as normalization of infective markers. The patient continued to receive intravenous ampicillin-sulbactam for a total duration of 6 weeks. A follow-up ultrasound scan after completion of antibiotic therapy revealed that the subcapsular perinephric collection and renal abscesses had completely resolved.

## DISCUSSION

Enterococci are gram-positive, catalase-negative, non-spore-forming, facultative anaerobic bacteria. They are important opportunistic pathogens that are becoming more widely recognized as a major cause of community-acquired and nosocomial infections. To date, more than 50 enterococci species have been identified, with *E. faecalis* and *E. faecium* being the most commonly cultured from humans [4]. Infections caused by *E. hirae* are common in animals but human infections are relatively rare. Pyelonephritis, biliary tract infections and infective endocarditis due to *E. hirae* have previously been reported in humans [5–8].

*E. hirae* infection occurs more commonly among patients with diabetes mellitus, liver cirrhosis, and chronic kidney disease [5]. Our patient had poorly controlled diabetes mellitus which could have predisposed to urinary tract infection caused by *E. hirae*. There are several case reports on *E. hirae* causing urinary tract infection. Oh *et al*. reported a case of *E. hirae* urinary tract infection in an elderly man with benign prostate hyperplasia. The patient was found to have acute pyelonephritis and acute kidney injury [6]. Chan *et al*. reported the first case of *E. hirae-*related acute pyelonephritis with bacteremia in a Taiwanese woman [7]. To our knowledge, this is the first case of *E. hirae* bacteremia causing subcapsular perinephric collection and renal abscesses.

According to published reports, human infection with *E. hirae* is extremely rare, which could be due to the difficulty in isolating *E. hirae* from clinical specimens using standard diagnostic methods [9]. MALDI-TOF MS is a useful tool for identifying Enterococcus species from wild birds and distinguishing between similar Enterococcus species [10]. Before the advent of MALDI-TOF MS, *E. hirae* may be underdiagnosed due to limitations of the diagnostic methods.

In conclusion, we report the first case of perinephric abscess and renal abscesses associated with *E. hirae* bacteremia using MALDI-TOF MS. The number of reported cases of *E. hirae* is expected to rise as MALDI-TOF MS becomes more widely used, so clinicians should consider *E. hirae* as a causative pathogen for urinary tract infections in susceptible patients.

## ABBREVIATIONS

DKA, Diabetic ketoacidosis CT, Computed tomography; MALDI-TOF MS, Matrix-assisted laser desorption/ionization-time of flight mass spectrometry

## References

[1] García-Solache M , Rice LB. The enterococcus: a model of adaptability to its environment. Clin Microbiol Rev 2019;32:e00058–18. 30700430 10.1128/CMR.00058-18 PMC6431128

[2] Bourafa N , Loucif L, Boutefnouchet N, Rolain JM. *Enterococcus hirae*, an unusual pathogen in humans causing urinary tract infection in a patient with benign prostatic hyperplasia: first case report in Algeria. New Microbes New Infect 2015;8:7–9. 26543562 10.1016/j.nmni.2015.08.003 PMC4590716

[3] Dicpinigaitis PV , De Aguirre M, Divito J. *Enterococcus hirae* Bacteremia associated with acute pancreatitis and septic shock. Case Rep Infect Dis 2015;2015:123852. 26417465 10.1155/2015/123852 PMC4568352

[4] Ramos S , Silva V, Dapkevicius MLE, Igrejas G, Poeta P. Enterococci, from harmless bacteria to a pathogen. Microorganisms 2020;8:1118. 10.3390/microorganisms8081118 PMC7463792 32722391

[5] Nakamura T , Ishikawa K, Matsuo T, Kawai F, Uehara Y, Mori N. *Enterococcus hirae* bacteremia associated with acute pyelonephritis in a patient with alcoholic cirrhosis: a case report and literature review. BMC Infect Dis 2021;21:999. 34556047 10.1186/s12879-021-06707-2 PMC8461981

[6] Oh JH , Cho AY, Kim YS, Lee KY, Sun IO. A rare case of urinary tract infection caused by *Enterococcus hirae* in an elderly man with benign prostate hyperplasia. Chonnam Med J 2019;55:177–8. 31598480 10.4068/cmj.2019.55.3.177 PMC6769244

[7] Chan TS , Wu MS, Suk FM, Chen CN, Chen YF, Hou YH, et al. *Enterococcus hirae*-related acute pyelonephritis and cholangitis with bacteremia: an unusual infection in humans. Kaohsiung J Med Sci 2012;28:111–4. 22313539 10.1016/j.kjms.2011.06.027 PMC11916197

[8] Poyart C , Lambert T, Morand P, Abassade P, Quesne G, Baudouy Y, et al. Native valve endocarditis due to *Enterococcus hirae*. J Clin Microbiol 2002;40:2689–90. 12089310 10.1128/JCM.40.7.2689-2690.2002 PMC120601

[9] Bilek HC , Deveci A, Ünal S, Tanrıverdi Çaycı Y, Tanyel E. *Enterococcus hirae* as a cause of bacteremic urinary tract infection: first case report from Turkey. J Infect Dev Ctries 2020;14:1780–482. 33378295 10.3855/jidc.12522

[10] Stępień-Pyśniak D , Hauschild T, Różański P, Marek A. MALDI-TOF mass spectrometry as a useful tool for identification of *Enterococcus* spp. from wild birds and differentiation of closely related species. J Microbiol Biotechnol 2017;27:1128–37. 28285496 10.4014/jmb.1612.12036

